# Diiodidobis(triphenyl­phosphine oxide)cadmium

**DOI:** 10.1107/S160053681005302X

**Published:** 2010-12-24

**Authors:** R. Shanthakumari, R. Hema, K. Ramamurthy, Helen Stoeckli-Evans

**Affiliations:** a Department of Physics, Government Arts College for Women, Pudukottai 622 001, Tamil Nadu, India; b Department of Physics, Seethalakshmi Ramaswami College (Autonomous), Tiruchirappalli 620 002, Tamil Nadu, India; c Crystal Growth and Thin Film Laboratory, School of Physics,, Bharathidasan University, Tiruchirapalli 620 024, India; d Institute of Physics, University of Neuchâtel, Rue Emile-Argand 11, CH-2009 Neuchâtel, Switzerland

## Abstract

In the title compound, [CdI_2_{(C_6_H_5_)_3_PO}_2_], the Cd^II^ atom is ligated by two I atoms and two O atoms from two triphenyl­phosphine oxide ligands in a disorted tetra­hedral arrangement. While the O—Cd—I angles vary from 106.67 (7) to 111.23 (7)°, the O—Cd—O angle is 88.60 (10)° and the I—Cd—I angle angle is 125.47 (2)°. The crystal structure is stabilized by van der Waals forces only.

## Related literature

For the structures of similar diiodo-bis­(triphenyl­phosphine oxide)–metal complexes, see: Beagley *et al.* (1988 ▶); Aviles *et al.* (1990 ▶); Cotton *et al.* (2002 ▶); Nie *et al.* (2005 ▶). For details of the Cambridge Structural Database, see: Allen (2002 ▶). 
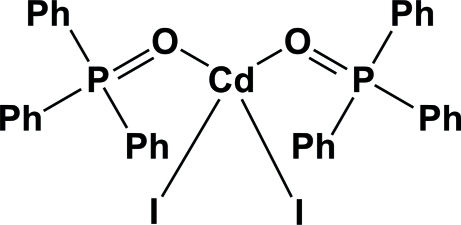


## Experimental

### 

#### Crystal data

[CdI_2_(C_18_H_15_OP)_2_]
*M* *_r_* = 922.74
Orthorhombic, 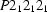

*a* = 10.5492 (5) Å
*b* = 17.7053 (7) Å
*c* = 19.1985 (9) Å
*V* = 3585.8 (3) Å^3^
*Z* = 4
Mo *K*α radiation
μ = 2.45 mm^−1^
*T* = 293 K
0.34 × 0.33 × 0.23 mm

#### Data collection

Stoe IPDS 2 diffractometer
Absorption correction: multi-scan (*MULscanABS* in *PLATON*; Spek, 2009 ▶) *T*_min_ = 0.786, *T*_max_ = 1.000
33377 measured reflections
6767 independent reflections
5935 reflections with *I* > 2σ(*I*)
*R*_int_ = 0.052

#### Refinement

*R*[*F* ^2^ > 2σ(*F* ^2^)] = 0.031
*wR*(*F* ^2^) = 0.050
*S* = 1.01
6767 reflections
389 parameters
H-atom parameters constrained
Δρ_max_ = 0.39 e Å^−3^
Δρ_min_ = −0.42 e Å^−3^
Absolute structure: Flack (1983 ▶), 2970 Friedel pairs
Flack parameter: −0.020 (16)

### 

Data collection: *X-AREA* (Stoe & Cie, 2009 ▶); cell refinement: *X-AREA*; data reduction: *X-RED32* (Stoe & Cie, 2009 ▶); program(s) used to solve structure: *SHELXS97* (Sheldrick, 2008 ▶); program(s) used to refine structure: *SHELXL97* (Sheldrick, 2008 ▶); molecular graphics: *PLATON* (Spek, 2009 ▶); software used to prepare material for publication: *SHELXL97* and *PLATON* (Spek, 2009 ▶).

## Supplementary Material

Crystal structure: contains datablocks I, global. DOI: 10.1107/S160053681005302X/hg2772sup1.cif

Structure factors: contains datablocks I. DOI: 10.1107/S160053681005302X/hg2772Isup2.hkl

Additional supplementary materials:  crystallographic information; 3D view; checkCIF report

## Figures and Tables

**Fig. 1.**
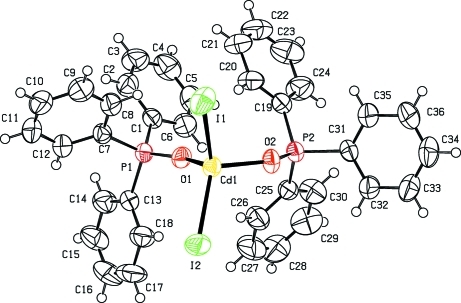
View of the molecular structure of the title compound, showing the atom-labelling scheme. Displacement ellipsoids are drawn at the 50% probability level.

**Fig. 2.**
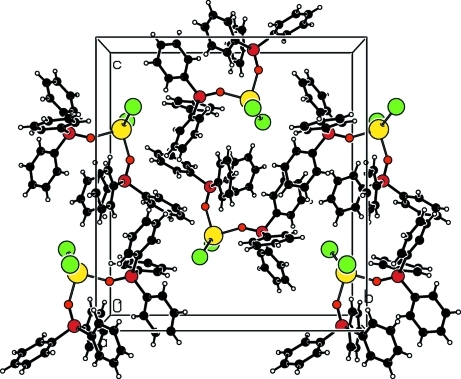
Crystal packing, viewed down a axis, of the title compound.

## supplementary crystallographic information

### Comment

The title compound consists of discrete {Cd[(C_6_H_5_)_3_PO]_2_I_2_] molecules (Fig. 1). It crystallized in the chiral orthorhombic space group *P*2_1_2_1_2_1_. Each cadmium atom is ligated by two iodine atoms and two oxygen atoms, of the triphenylphosphine oxide ligands, which yield an approximate tetrahedral coordination geometry.

A search of the Cambridge Structural Database (CSD, version 5.31, last update Aug. 2010; Allen, 2002) gave 13 hits for non-solvated *M*[(Ph)_3_PO]_2_*X*_2_ complexes (where *M* = Mn, Co, Ni, Cu, Zn, Hg; and *X* = Cl, Br, I), all of which crystallized in non-centrosymmetric space groups; Fdd2 (5×), P1(5×), *Cc*(1×), Pca21(1×) and Pna21(1×). The iodide complexes, which include *M* = Mn (Beagley *et al.*, 1988; Aviles *et al.*, 1990), *M* = Co (Cotton *et al.*, 2002) and *M* = Zn (Nie *et al.*, 2005), all crystallized in the noncentrosymmetric triclinic space group P1. The title compound is the first cadmium halide complex to be obtained and the first that crystallizes in space group *P*2_1_2_1_2_1_.

The coordination polyhedron of the title compound is considerabley distorted. While the I—Cd—O angles vary from 106.67 (7) to 111.23 (7) °, angle O1—Cd1—O2 is 88.6 (1) ° and angle I1—Cd1—I2 angle is 125.47 (2) °. This differs significantly from the situation in the three iodide complexes mentioned above. There the O···M···O angles vary from 101.0 - 105.2 ° and the I—M—I angles from 112.5 - 116.3 °.

In the crystal there are no π···π stacking interactions, the molecules are simply stabilized by Van der Waals forces (Fig. 2).

### Experimental

The title compound was synthesized by reacting triphenylphosphine oxide and cadmium iodide in a 1:1 (1.8 g:2.5 g) molar ratio. The calculated amount of triphenylphosphine oxide was dissolved in ethanol and the cadmium iodide was slowly added to the solution with stirring. Within a few minutes the solution became turbid. The reaction was ensured with continuous strirring. After an hour the product, a white salt, deposited at the bottom of the beaker and was then filtered and dried. This compound was recrystallized from DMSO to give colourless block-like crystals of title compound.

### Refinement

The C-bound H-atoms were included in calculated positions and treated as riding atoms: C—H = 0.93 Å, with *U*_iso_(H) = 1.2 *U*_eq_(parent C-atom).

### Crystal data

**Table:**

[CdI_2_(C_18_H_15_OP)_2_]	*F*(000) = 1784
*M**_r_* = 922.74	*D*_x_ = 1.709 Mg m^−^^3^
Orthorhombic, *P*2_1_2_1_2_1_	Mo *K*α radiation, λ = 0.71073 Å
Hall symbol: P 2ac 2ab	Cell parameters from 24384 reflections
*a* = 10.5492 (5) Å	θ = 1.6–26.1°
*b* = 17.7053 (7) Å	µ = 2.45 mm^−^^1^
*c* = 19.1985 (9) Å	*T* = 293 K
*V* = 3585.8 (3) Å^3^	Block, colourless
*Z* = 4	0.34 × 0.33 × 0.23 mm

### Data collection

**Table:**

Stoe IPDS 2 diffractometer	6767 independent reflections
Radiation source: fine-focus sealed tube	5935 reflections with *I* > 2σ(*I*)
graphite	*R*_int_ = 0.052
φ and ω scans	θ_max_ = 25.7°, θ_min_ = 1.6°
Absorption correction: multi-scan (MULscanABS on *PLATON*; Spek, 2009)	*h* = −12→12
*T*_min_ = 0.786, *T*_max_ = 1.000	*k* = −21→21
33377 measured reflections	*l* = −23→22

### Refinement

**Table:**

Refinement on *F*^2^	Hydrogen site location: inferred from neighbouring sites
Least-squares matrix: full	H-atom parameters constrained
*R*[*F*^2^ > 2σ(*F*^2^)] = 0.031	*w* = 1/[σ^2^(*F*_o_^2^) + (0.0223*P*)^2^] where *P* = (*F*_o_^2^ + 2*F*_c_^2^)/3
*wR*(*F*^2^) = 0.050	(Δ/σ)_max_ = 0.001
*S* = 1.01	Δρ_max_ = 0.39 e Å^−^^3^
6767 reflections	Δρ_min_ = −0.42 e Å^−^^3^
389 parameters	Extinction correction: *SHELXL97* (Sheldrick, 2008), Fc^*^=kFc[1+0.001xFc^2^λ^3^/sin(2θ)]^-1/4^
0 restraints	Extinction coefficient: 0.00162 (7)
Primary atom site location: structure-invariant direct methods	Absolute structure: Flack (1983), 2950 Friedel pairs
Secondary atom site location: difference Fourier map	Flack parameter: −0.020 (16)

### Special details

**Table:**

Geometry. All e.s.d.'s (except the e.s.d. in the dihedral angle between two l.s. planes) are estimated using the full covariance matrix. The cell e.s.d.'s are taken into account individually in the estimation of e.s.d.'s in distances, angles and torsion angles; correlations between e.s.d.'s in cell parameters are only used when they are defined by crystal symmetry. An approximate (isotropic) treatment of cell e.s.d.'s is used for estimating e.s.d.'s involving l.s. planes.
Refinement. Refinement of *F*^2^ against ALL reflections. The weighted *R*-factor *wR* and goodness of fit *S* are based on *F*^2^, conventional *R*-factors *R* are based on *F*, with *F* set to zero for negative *F*^2^. The threshold expression of *F*^2^ > σ(*F*^2^) is used only for calculating *R*-factors(gt) *etc*. and is not relevant to the choice of reflections for refinement. *R*-factors based on *F*^2^ are statistically about twice as large as those based on *F*, and *R*- factors based on ALL data will be even larger.

### Fractional atomic coordinates and isotropic or equivalent isotropic displacement parameters (Å^2^)

**Table:**

	*x*	*y*	*z*	*U*_iso_*/*U*_eq_	
I1	1.18644 (3)	−0.082133 (18)	0.23181 (2)	0.07658 (11)	
I2	0.74406 (3)	−0.126641 (17)	0.270473 (19)	0.06842 (10)	
Cd1	0.93992 (3)	−0.072930 (15)	0.195467 (15)	0.04256 (8)	
P1	0.86596 (10)	0.12527 (5)	0.18071 (5)	0.0410 (2)	
P2	0.90645 (9)	−0.08668 (5)	0.01070 (5)	0.0403 (2)	
O1	0.8885 (3)	0.04392 (13)	0.16396 (15)	0.0484 (7)	
O2	0.9237 (3)	−0.10704 (13)	0.08553 (14)	0.0487 (7)	
C1	0.9126 (4)	0.1857 (2)	0.1099 (2)	0.0453 (10)	
C2	0.9962 (5)	0.2454 (2)	0.1185 (3)	0.0598 (12)	
H2	1.0317	0.2553	0.1619	0.072*	
C3	1.0259 (5)	0.2898 (3)	0.0619 (3)	0.0737 (15)	
H3	1.0807	0.3305	0.0680	0.088*	
C4	0.9779 (6)	0.2762 (3)	−0.0025 (3)	0.0769 (16)	
H4	0.9999	0.3069	−0.0398	0.092*	
C5	0.8971 (5)	0.2169 (3)	−0.0117 (3)	0.0747 (15)	
H5	0.8638	0.2070	−0.0557	0.090*	
C6	0.8643 (5)	0.1713 (3)	0.0443 (3)	0.0627 (12)	
H6	0.8095	0.1308	0.0377	0.075*	
C7	0.9530 (4)	0.15302 (18)	0.2570 (2)	0.0438 (10)	
C8	1.0737 (4)	0.1233 (2)	0.2657 (3)	0.0610 (11)	
H8	1.1075	0.0917	0.2318	0.073*	
C9	1.1433 (5)	0.1401 (3)	0.3236 (3)	0.0762 (15)	
H9	1.2230	0.1185	0.3296	0.091*	
C10	1.0953 (5)	0.1895 (3)	0.3737 (3)	0.0755 (15)	
H10	1.1425	0.2008	0.4132	0.091*	
C11	0.9782 (5)	0.2212 (3)	0.3643 (3)	0.0692 (15)	
H11	0.9465	0.2551	0.3969	0.083*	
C12	0.9073 (4)	0.2027 (2)	0.3063 (3)	0.0555 (11)	
H12	0.8276	0.2242	0.3004	0.067*	
C13	0.7004 (4)	0.1429 (2)	0.1953 (2)	0.0455 (10)	
C14	0.6512 (4)	0.2156 (3)	0.1886 (3)	0.0641 (13)	
H14	0.7052	0.2555	0.1779	0.077*	
C15	0.5250 (5)	0.2291 (3)	0.1974 (3)	0.0776 (15)	
H15	0.4934	0.2778	0.1926	0.093*	
C16	0.4463 (5)	0.1718 (3)	0.2132 (3)	0.0846 (18)	
H16	0.3600	0.1811	0.2180	0.101*	
C17	0.4920 (5)	0.0997 (3)	0.2223 (3)	0.0905 (18)	
H17	0.4374	0.0607	0.2347	0.109*	
C18	0.6202 (4)	0.0855 (3)	0.2127 (3)	0.0662 (13)	
H18	0.6515	0.0367	0.2181	0.079*	
C19	1.0159 (4)	−0.0149 (2)	−0.0169 (2)	0.0471 (10)	
C20	1.0988 (4)	0.0163 (3)	0.0302 (3)	0.0639 (13)	
H20	1.1012	−0.0021	0.0756	0.077*	
C21	1.1794 (5)	0.0753 (3)	0.0112 (3)	0.0829 (16)	
H21	1.2331	0.0968	0.0441	0.099*	
C22	1.1795 (5)	0.1011 (3)	−0.0550 (4)	0.0827 (17)	
H22	1.2346	0.1396	−0.0680	0.099*	
C23	1.0993 (6)	0.0709 (3)	−0.1023 (4)	0.0899 (18)	
H23	1.0994	0.0892	−0.1477	0.108*	
C24	1.0169 (6)	0.0132 (3)	−0.0843 (3)	0.0771 (16)	
H24	0.9622	−0.0068	−0.1175	0.093*	
C25	0.7489 (4)	−0.0529 (2)	−0.0065 (2)	0.0454 (9)	
C26	0.6703 (5)	−0.0353 (3)	0.0477 (3)	0.0666 (13)	
H26	0.6986	−0.0412	0.0933	0.080*	
C27	0.5483 (5)	−0.0085 (3)	0.0355 (4)	0.0921 (19)	
H27	0.4955	0.0032	0.0728	0.111*	
C28	0.5063 (6)	0.0007 (3)	−0.0305 (4)	0.0869 (19)	
H28	0.4249	0.0189	−0.0385	0.104*	
C29	0.5831 (6)	−0.0168 (3)	−0.0857 (4)	0.0833 (17)	
H29	0.5536	−0.0107	−0.1310	0.100*	
C30	0.7039 (5)	−0.0433 (3)	−0.0743 (3)	0.0694 (14)	
H30	0.7559	−0.0549	−0.1119	0.083*	
C31	0.9299 (4)	−0.1687 (2)	−0.0436 (2)	0.0450 (9)	
C32	0.8330 (5)	−0.2208 (2)	−0.0492 (3)	0.0586 (12)	
H32	0.7548	−0.2109	−0.0288	0.070*	
C33	0.8515 (5)	−0.2872 (3)	−0.0847 (3)	0.0704 (15)	
H33	0.7859	−0.3220	−0.0885	0.085*	
C34	0.9669 (5)	−0.3021 (3)	−0.1145 (3)	0.0752 (16)	
H34	0.9792	−0.3470	−0.1386	0.090*	
C35	1.0644 (7)	−0.2510 (3)	−0.1091 (3)	0.0888 (18)	
H35	1.1423	−0.2608	−0.1299	0.107*	
C36	1.0455 (5)	−0.1847 (3)	−0.0721 (3)	0.0694 (14)	
H36	1.1121	−0.1508	−0.0666	0.083*	

### Atomic displacement parameters (Å^2^)

**Table:**

	*U*^11^	*U*^22^	*U*^33^	*U*^12^	*U*^13^	*U*^23^
I1	0.06132 (19)	0.06743 (18)	0.1010 (3)	0.01232 (16)	−0.03074 (19)	−0.0126 (2)
I2	0.0711 (2)	0.06905 (19)	0.0651 (2)	−0.01968 (16)	0.01331 (18)	0.00869 (16)
Cd1	0.04879 (16)	0.04223 (14)	0.03666 (15)	−0.00255 (14)	−0.00130 (13)	−0.00194 (13)
P1	0.0430 (6)	0.0347 (5)	0.0454 (7)	0.0008 (4)	0.0039 (5)	−0.0052 (4)
P2	0.0476 (6)	0.0388 (5)	0.0345 (5)	0.0016 (4)	0.0015 (4)	−0.0018 (4)
O1	0.0506 (17)	0.0352 (13)	0.0595 (18)	0.0008 (11)	0.0012 (15)	−0.0045 (12)
O2	0.0673 (18)	0.0434 (13)	0.0355 (16)	0.0021 (14)	−0.0002 (14)	−0.0049 (11)
C1	0.046 (3)	0.044 (2)	0.046 (3)	0.0039 (19)	0.008 (2)	−0.0018 (19)
C2	0.068 (3)	0.053 (3)	0.058 (3)	−0.008 (2)	0.003 (3)	−0.003 (2)
C3	0.078 (4)	0.060 (3)	0.084 (4)	−0.016 (2)	0.017 (3)	0.013 (3)
C4	0.092 (4)	0.080 (3)	0.059 (4)	0.001 (3)	0.018 (3)	0.018 (3)
C5	0.085 (4)	0.086 (4)	0.052 (3)	0.008 (3)	0.001 (3)	0.000 (3)
C6	0.070 (3)	0.057 (3)	0.062 (3)	−0.002 (2)	0.006 (3)	0.001 (2)
C7	0.050 (2)	0.0379 (18)	0.043 (3)	−0.0009 (17)	0.005 (2)	−0.0016 (17)
C8	0.054 (3)	0.062 (2)	0.067 (3)	0.002 (2)	0.002 (3)	−0.015 (3)
C9	0.054 (3)	0.098 (4)	0.076 (4)	−0.002 (3)	−0.014 (3)	−0.012 (3)
C10	0.070 (4)	0.091 (4)	0.065 (4)	−0.019 (3)	−0.002 (3)	−0.015 (3)
C11	0.078 (4)	0.074 (3)	0.056 (3)	−0.012 (3)	0.004 (3)	−0.022 (3)
C12	0.058 (3)	0.051 (2)	0.057 (3)	0.000 (2)	0.004 (2)	−0.011 (2)
C13	0.046 (2)	0.046 (2)	0.044 (2)	0.0018 (17)	0.005 (2)	−0.0054 (19)
C14	0.057 (3)	0.057 (3)	0.079 (4)	0.007 (2)	0.009 (3)	−0.001 (3)
C15	0.065 (4)	0.076 (3)	0.092 (4)	0.020 (3)	0.021 (3)	0.007 (3)
C16	0.053 (3)	0.109 (4)	0.092 (5)	0.028 (3)	0.019 (3)	0.002 (3)
C17	0.059 (3)	0.097 (4)	0.116 (5)	−0.019 (3)	0.037 (3)	0.004 (3)
C18	0.059 (3)	0.058 (2)	0.082 (4)	−0.004 (2)	0.019 (2)	0.001 (2)
C19	0.049 (3)	0.047 (2)	0.045 (3)	0.0030 (19)	0.007 (2)	−0.0001 (19)
C20	0.047 (3)	0.074 (3)	0.070 (4)	−0.011 (2)	0.007 (3)	0.006 (3)
C21	0.056 (3)	0.086 (4)	0.107 (5)	−0.024 (3)	0.013 (3)	−0.010 (4)
C22	0.058 (3)	0.069 (3)	0.120 (6)	−0.009 (3)	0.017 (4)	0.019 (3)
C23	0.087 (4)	0.088 (4)	0.094 (4)	−0.001 (4)	0.022 (4)	0.039 (4)
C24	0.082 (4)	0.085 (4)	0.065 (4)	−0.022 (3)	0.004 (3)	0.013 (3)
C25	0.047 (2)	0.040 (2)	0.049 (3)	0.0003 (18)	0.006 (2)	0.0006 (17)
C26	0.056 (3)	0.090 (3)	0.054 (3)	0.005 (3)	0.001 (3)	−0.010 (3)
C27	0.046 (3)	0.121 (5)	0.110 (6)	0.019 (3)	0.005 (3)	−0.019 (4)
C28	0.056 (4)	0.077 (4)	0.127 (6)	0.021 (3)	−0.021 (4)	−0.007 (4)
C29	0.070 (4)	0.084 (4)	0.096 (5)	0.014 (3)	−0.023 (4)	0.023 (3)
C30	0.061 (3)	0.085 (3)	0.062 (3)	0.011 (3)	−0.003 (3)	0.010 (3)
C31	0.048 (2)	0.051 (2)	0.036 (2)	0.008 (2)	0.001 (2)	−0.0058 (18)
C32	0.057 (3)	0.056 (3)	0.063 (3)	0.002 (2)	0.000 (2)	−0.012 (2)
C33	0.075 (4)	0.054 (3)	0.083 (4)	0.002 (3)	−0.016 (3)	−0.019 (3)
C34	0.092 (5)	0.057 (3)	0.078 (4)	0.017 (3)	−0.011 (3)	−0.022 (3)
C35	0.074 (4)	0.086 (4)	0.107 (5)	0.014 (4)	0.020 (4)	−0.040 (3)
C36	0.066 (3)	0.063 (3)	0.079 (4)	−0.002 (3)	0.009 (3)	−0.023 (3)

### Geometric parameters (Å, °)

**Table:**

I1—Cd1	2.6975 (4)	C15—H15	0.9300
I2—Cd1	2.6920 (4)	C16—C17	1.376 (7)
Cd1—O2	2.202 (3)	C16—H16	0.9300
Cd1—O1	2.223 (2)	C17—C18	1.388 (7)
P1—O1	1.495 (3)	C17—H17	0.9300
P1—C13	1.796 (4)	C18—H18	0.9300
P1—C7	1.797 (4)	C19—C20	1.375 (6)
P1—C1	1.799 (4)	C19—C24	1.386 (6)
P2—O2	1.492 (3)	C20—C21	1.395 (7)
P2—C25	1.796 (5)	C20—H20	0.9300
P2—C19	1.797 (4)	C21—C22	1.350 (8)
P2—C31	1.805 (4)	C21—H21	0.9300
C1—C6	1.381 (6)	C22—C23	1.352 (8)
C1—C2	1.385 (6)	C22—H22	0.9300
C2—C3	1.379 (7)	C23—C24	1.385 (7)
C2—H2	0.9300	C23—H23	0.9300
C3—C4	1.357 (8)	C24—H24	0.9300
C3—H3	0.9300	C25—C26	1.367 (6)
C4—C5	1.364 (7)	C25—C30	1.397 (6)
C4—H4	0.9300	C26—C27	1.391 (7)
C5—C6	1.389 (7)	C26—H26	0.9300
C5—H5	0.9300	C27—C28	1.352 (8)
C6—H6	0.9300	C27—H27	0.9300
C7—C12	1.379 (5)	C28—C29	1.369 (8)
C7—C8	1.389 (6)	C28—H28	0.9300
C8—C9	1.364 (7)	C29—C30	1.375 (7)
C8—H8	0.9300	C29—H29	0.9300
C9—C10	1.396 (7)	C30—H30	0.9300
C9—H9	0.9300	C31—C36	1.366 (6)
C10—C11	1.368 (8)	C31—C32	1.382 (6)
C10—H10	0.9300	C32—C33	1.372 (6)
C11—C12	1.380 (7)	C32—H32	0.9300
C11—H11	0.9300	C33—C34	1.370 (7)
C12—H12	0.9300	C33—H33	0.9300
C13—C18	1.364 (6)	C34—C35	1.374 (7)
C13—C14	1.393 (6)	C34—H34	0.9300
C14—C15	1.363 (7)	C35—C36	1.387 (6)
C14—H14	0.9300	C35—H35	0.9300
C15—C16	1.345 (7)	C36—H36	0.9300
			
O2—Cd1—O1	88.60 (10)	C14—C15—H15	120.0
O2—Cd1—I2	110.88 (8)	C15—C16—C17	120.7 (5)
O1—Cd1—I2	106.67 (7)	C15—C16—H16	119.6
O2—Cd1—I1	107.82 (8)	C17—C16—H16	119.6
O1—Cd1—I1	111.23 (7)	C16—C17—C18	119.5 (5)
I2—Cd1—I1	125.469 (16)	C16—C17—H17	120.2
O1—P1—C13	110.84 (17)	C18—C17—H17	120.2
O1—P1—C7	110.96 (17)	C13—C18—C17	120.1 (5)
C13—P1—C7	108.8 (2)	C13—C18—H18	120.0
O1—P1—C1	111.55 (17)	C17—C18—H18	120.0
C13—P1—C1	106.26 (19)	C20—C19—C24	117.7 (4)
C7—P1—C1	108.28 (19)	C20—C19—P2	119.9 (3)
O2—P2—C25	111.72 (19)	C24—C19—P2	122.3 (4)
O2—P2—C19	112.11 (19)	C19—C20—C21	121.1 (5)
C25—P2—C19	107.76 (19)	C19—C20—H20	119.5
O2—P2—C31	110.16 (16)	C21—C20—H20	119.5
C25—P2—C31	106.78 (19)	C22—C21—C20	120.0 (5)
C19—P2—C31	108.1 (2)	C22—C21—H21	120.0
P1—O1—Cd1	151.30 (18)	C20—C21—H21	120.0
P2—O2—Cd1	149.99 (15)	C21—C22—C23	119.8 (5)
C6—C1—C2	119.0 (4)	C21—C22—H22	120.1
C6—C1—P1	118.5 (3)	C23—C22—H22	120.1
C2—C1—P1	122.5 (4)	C22—C23—C24	121.2 (6)
C3—C2—C1	119.1 (5)	C22—C23—H23	119.4
C3—C2—H2	120.5	C24—C23—H23	119.4
C1—C2—H2	120.5	C23—C24—C19	120.1 (5)
C4—C3—C2	122.1 (5)	C23—C24—H24	119.9
C4—C3—H3	119.0	C19—C24—H24	119.9
C2—C3—H3	119.0	C26—C25—C30	118.4 (4)
C3—C4—C5	119.3 (5)	C26—C25—P2	119.9 (4)
C3—C4—H4	120.4	C30—C25—P2	121.7 (3)
C5—C4—H4	120.4	C25—C26—C27	120.7 (5)
C4—C5—C6	120.1 (5)	C25—C26—H26	119.7
C4—C5—H5	120.0	C27—C26—H26	119.7
C6—C5—H5	120.0	C28—C27—C26	120.2 (6)
C1—C6—C5	120.5 (5)	C28—C27—H27	119.9
C1—C6—H6	119.8	C26—C27—H27	119.9
C5—C6—H6	119.8	C27—C28—C29	120.3 (5)
C12—C7—C8	118.7 (4)	C27—C28—H28	119.9
C12—C7—P1	123.8 (3)	C29—C28—H28	119.9
C8—C7—P1	117.6 (3)	C28—C29—C30	120.2 (6)
C9—C8—C7	120.6 (5)	C28—C29—H29	119.9
C9—C8—H8	119.7	C30—C29—H29	119.9
C7—C8—H8	119.7	C29—C30—C25	120.3 (5)
C8—C9—C10	120.1 (5)	C29—C30—H30	119.8
C8—C9—H9	119.9	C25—C30—H30	119.8
C10—C9—H9	119.9	C36—C31—C32	119.4 (4)
C11—C10—C9	119.6 (5)	C36—C31—P2	121.4 (3)
C11—C10—H10	120.2	C32—C31—P2	118.8 (3)
C9—C10—H10	120.2	C33—C32—C31	120.3 (5)
C10—C11—C12	119.9 (5)	C33—C32—H32	119.8
C10—C11—H11	120.1	C31—C32—H32	119.8
C12—C11—H11	120.1	C34—C33—C32	119.9 (5)
C11—C12—C7	121.0 (4)	C34—C33—H33	120.0
C11—C12—H12	119.5	C32—C33—H33	120.0
C7—C12—H12	119.5	C33—C34—C35	120.5 (5)
C18—C13—C14	118.7 (4)	C33—C34—H34	119.8
C18—C13—P1	120.7 (3)	C35—C34—H34	119.8
C14—C13—P1	120.6 (3)	C34—C35—C36	119.2 (6)
C15—C14—C13	120.9 (5)	C34—C35—H35	120.4
C15—C14—H14	119.6	C36—C35—H35	120.4
C13—C14—H14	119.6	C31—C36—C35	120.6 (5)
C16—C15—C14	120.0 (5)	C31—C36—H36	119.7
C16—C15—H15	120.0	C35—C36—H36	119.7
			
C13—P1—O1—Cd1	−95.8 (4)	C13—C14—C15—C16	0.3 (9)
C7—P1—O1—Cd1	25.2 (4)	C14—C15—C16—C17	1.6 (10)
C1—P1—O1—Cd1	146.0 (3)	C15—C16—C17—C18	−2.2 (10)
O2—Cd1—O1—P1	−177.4 (4)	C14—C13—C18—C17	1.0 (7)
I2—Cd1—O1—P1	71.2 (4)	P1—C13—C18—C17	−178.6 (5)
I1—Cd1—O1—P1	−68.8 (4)	C16—C17—C18—C13	0.9 (9)
C25—P2—O2—Cd1	−69.3 (4)	O2—P2—C19—C20	−2.5 (4)
C19—P2—O2—Cd1	51.8 (4)	C25—P2—C19—C20	120.8 (4)
C31—P2—O2—Cd1	172.2 (3)	C31—P2—C19—C20	−124.1 (4)
O1—Cd1—O2—P2	7.7 (4)	O2—P2—C19—C24	−179.7 (4)
I2—Cd1—O2—P2	115.0 (4)	C25—P2—C19—C24	−56.3 (5)
I1—Cd1—O2—P2	−104.2 (4)	C31—P2—C19—C24	58.7 (5)
O1—P1—C1—C6	53.1 (4)	C24—C19—C20—C21	1.2 (7)
C13—P1—C1—C6	−67.8 (4)	P2—C19—C20—C21	−176.1 (4)
C7—P1—C1—C6	175.4 (3)	C19—C20—C21—C22	−1.9 (8)
O1—P1—C1—C2	−126.1 (4)	C20—C21—C22—C23	1.5 (9)
C13—P1—C1—C2	113.0 (4)	C21—C22—C23—C24	−0.4 (9)
C7—P1—C1—C2	−3.7 (4)	C22—C23—C24—C19	−0.3 (9)
C6—C1—C2—C3	1.8 (7)	C20—C19—C24—C23	−0.1 (8)
P1—C1—C2—C3	−179.1 (4)	P2—C19—C24—C23	177.1 (4)
C1—C2—C3—C4	−1.3 (8)	O2—P2—C25—C26	12.2 (4)
C2—C3—C4—C5	0.3 (8)	C19—P2—C25—C26	−111.4 (4)
C3—C4—C5—C6	0.2 (8)	C31—P2—C25—C26	132.7 (4)
C2—C1—C6—C5	−1.3 (7)	O2—P2—C25—C30	−168.4 (4)
P1—C1—C6—C5	179.5 (4)	C19—P2—C25—C30	68.0 (4)
C4—C5—C6—C1	0.3 (7)	C31—P2—C25—C30	−47.9 (4)
O1—P1—C7—C12	−142.9 (3)	C30—C25—C26—C27	0.1 (7)
C13—P1—C7—C12	−20.7 (4)	P2—C25—C26—C27	179.5 (4)
C1—P1—C7—C12	94.4 (4)	C25—C26—C27—C28	−0.2 (9)
O1—P1—C7—C8	38.2 (4)	C26—C27—C28—C29	0.4 (10)
C13—P1—C7—C8	160.4 (3)	C27—C28—C29—C30	−0.4 (9)
C1—P1—C7—C8	−84.6 (3)	C28—C29—C30—C25	0.2 (8)
C12—C7—C8—C9	3.2 (7)	C26—C25—C30—C29	−0.1 (7)
P1—C7—C8—C9	−177.9 (4)	P2—C25—C30—C29	−179.5 (4)
C7—C8—C9—C10	−2.1 (8)	O2—P2—C31—C36	−93.3 (4)
C8—C9—C10—C11	−0.2 (8)	C25—P2—C31—C36	145.2 (4)
C9—C10—C11—C12	1.5 (8)	C19—P2—C31—C36	29.5 (4)
C10—C11—C12—C7	−0.5 (7)	O2—P2—C31—C32	78.7 (4)
C8—C7—C12—C11	−1.9 (6)	C25—P2—C31—C32	−42.8 (4)
P1—C7—C12—C11	179.2 (4)	C19—P2—C31—C32	−158.5 (3)
O1—P1—C13—C18	21.2 (4)	C36—C31—C32—C33	−1.8 (7)
C7—P1—C13—C18	−101.1 (4)	P2—C31—C32—C33	−174.0 (4)
C1—P1—C13—C18	142.5 (4)	C31—C32—C33—C34	0.3 (8)
O1—P1—C13—C14	−158.4 (4)	C32—C33—C34—C35	0.1 (9)
C7—P1—C13—C14	79.3 (4)	C33—C34—C35—C36	1.0 (10)
C1—P1—C13—C14	−37.0 (4)	C32—C31—C36—C35	2.9 (8)
C18—C13—C14—C15	−1.6 (8)	P2—C31—C36—C35	174.9 (5)
P1—C13—C14—C15	178.0 (5)	C34—C35—C36—C31	−2.5 (10)
